# The Impact of the COVID‐19 Pandemic on Nurses' and Junior Doctors' Workloads

**DOI:** 10.1111/jan.16487

**Published:** 2024-09-22

**Authors:** Gemma Doleman, Annemarie De Leo, Dianne Bloxsome

**Affiliations:** ^1^ School of Nursing and Midwifery Edith Cowan University Perth Western Australia Australia; ^2^ Centre for Nursing Research Sir Charles Gardiner Osbourne Park Healthcare Group Perth Western Australia Australia; ^3^ School of Medical Health Sciences Edith Cowan University Perth Western Australia Australia

**Keywords:** doctors, nurses, pandemic, workloads

## Abstract

**Aims:**

To explore the impact of the COVID‐19 pandemic on nurses' and junior doctors' workload, changes to direct care and the impact of workload on resource allocation.

**Design:**

Mixed‐method design was used.

**Methods:**

Data were collected from direct observation, hospital administrative database and a survey. Nurses and junior doctors were observed on two COVID‐19 wards and one non‐COVID‐19 ward using a semi‐structured observation schedule. Survey data were collected from nurses and junior doctors that had worked on the COVID‐19 ward during the pandemic. Patient data were collected from Web Patient Administration System during the period of March–August 2021 and compared to March–August 2022.

**Results:**

The results of this study indicated that the workloads of nurses and junior doctors changed on the COVID‐19 wards. Nurses on the COVID‐19 ward spent less time on administration, communication and documentation compared to nurses working on the non‐COVID‐19 ward. For junior doctors, less time was being spent on direct care activities and administrative work compared to the non‐COVID ward. On the COVID‐19 wards, patients were older and had a shorter length of stay compared to the year before. Five themes were identified from the surveys, staffing shortages resulting in higher workloads and the need for overtime, unforeseen time to undertake tasks, lack of support and physical health and patient care compromised.

**Conclusion:**

The study highlights the need for systemic changes to staffing shortages and elevated workloads to improve the compromised mental and physical health caused during the pandemic and to retain the workforce for future sustainability.

**Implications:**

As we collectively reflect on the lessons learned from this unprecedented period, it is imperative to address the challenges experienced proactively, fostering a healthcare environment that prioritises the well‐being of its front line heroes and, by extension, the quality of patient care.

**Reporting Method:**

STROBE.

**Patient or Public Contribution:**

No patient or public contribution.

**Summary**

- **What Problem Did the Study Address?**
  - To explore the impact of the COVID‐19 pandemic on nurses' and junior doctors' workload, changes to direct care and the impact of workload on resource allocation.
- **What Were the Main Findings?**
  - The workloads of nurses' and junior doctors' changed as a result of the pandemic. For nurses, this meant less time on administration, communication, and documentation and for junior doctors, less time on direct care activities and administrative work. This resulted to changes in patient care and mental health distress for the workforce.
- **Where and on Whom Will the Research Have an Impact?**
  - The results of this study shed light on the impact of the pandemic on workloads. These challenges need to be acknowledged and addressed to retain the workforce into the future and to prevent similar situations arising in future pandemics or emergency situations.

## 1 Introduction

In 2019, an unknown aetiology of Pneumonia was discovered in Wuhan City, China (Budisusilowati and Darmawan 2023). The World Health Organization (WHO) declared a public health emergency with the outbreak of Severe Acute Respiratory Syndrome Coronavirus 2 (SARS‐Co V‐2) in January 2020; in March, a global pandemic identified as COVID‐19 was confirmed (Sohrabi et al. 2020). The COVID‐19 pandemic profoundly impacted global health systems as healthcare organisations rapidly responded to the unfolding circumstances (Hiscott et al. 2020), re‐directing resources and reducing non‐essential healthcare activities (Murphy, Akehurst, and Mutimer 2020).

Healthcare workers were confronted with many challenges during the COVID‐19 outbreak (Yusefi et al. 2022); heavy workloads, increased patient acuity and heightened demands of work are reported on a global scale (Budisusilowati and Darmawan 2023; Koontalay et al. 2021; Yusefi et al. 2022). Healthcare workers were also faced with longer working hours, redeployment and precipitated burnout (Revythis et al. 2021). Nursing professionals are among the most severely affected (Souza 2021); reporting higher levels of stress, shifts in workload allocation compared with other front line workers (Budisusilowati and Darmawan 2023), job strain and increased patient‐to‐staff ratios (Sarafis et al. 2016). Published work also confirms doctors experienced similar occupational burdens: understaffing, increased workload and working beyond usual competencies (Spiers et al. 2021). Junior doctors were highlighted to report additional stressors such as managing multiple unwell patients, absenteeism of senior staff and extreme workload pressure (Coughlan et al. 2021).

The global COVID‐19 health crisis poses major operational and logistical challenges, even in high‐income countries and well‐resourced health systems (Coughlan et al. 2021). While health organisations have increased capacity and resources to meet the demands for care, nurses and junior doctors continue to work in high‐pressure environments where workloads remain an issue (Budisusilowati and Darmawan 2023; Spiers et al. 2021).

The concept of workload since the COVID‐19 outbreak has evolved significantly. Workload is traditionally recognised to encompass a set of dynamic factors that influence the work process, incorporating both internal and external environmental factors (Souza 2021). Additional variables have since been reported, including excessive working hours, constant patient demands, physical exertion and fear of contracting COVID‐19 for those in direct care roles (Christianson et al. 2022; Hiscott et al. 2020; Koontalay et al. 2021; Yusefi et al. 2022). To date, no literature specifically explores the impact of nurses' and junior doctors' workloads in relation to direct care duties and resource allocation during the COVID‐19 pandemic; how this has changed and the implications of such change on nurses and junior doctors' workloads. Therefore, the aim of this study was to explore the impact of the COVID‐19 pandemic on nurses' and junior doctors' workload, changes to direct care and the impact of workload on resource allocation.

## 2 Methods

### 2.1 Design and Setting

The study used an explanatory mixed‐method design, by collecting quantitative data through observations and qualitative data through an open‐ended survey. Supplementary patient data were collected from the hospital administrative database Web Patient Administration System (webPAS). By using quantitative and qualitative data sources together, a greater understanding and insight into the workload changes experienced by nurses and junior doctors during the pandemic can be obtained (Bowen, Rose, and Pilkington 2017). Nurses and junior doctors working on both COVID‐only wards and non‐COVID wards in one public hospital in Western Australia (WA) were observed. For this study, a COVID‐19 ward was defined as any ward exclusively looking after COVID‐19‐positive patients. A non‐COVID‐19 ward was defined as a ward not allocated COVID‐19‐positive patients. Convenience sampling was used in that all nurses and junior doctors working on the COVID and non‐COVID wards were invited to participate.

### 2.2 Data Collection

Data collection took place across three wards at the study site between May 2022 and January 2023. Two of the wards were defined as COVID‐19 only and one as non‐COVID‐19. Data collection involved nurses and junior doctor observations, a review of patient records and an open‐ended survey. Two research assistants were trained in non‐participation observation audits for the study. Each observation session lasted 2 h in which the same nurse or junior doctor was observed for the full 2‐h time period. All data were collected during normal working hours. Due to the nature of the study and shift work, it was possible that a nurse or doctor be observed more than once during the study period. All observed activities, including both direct and non‐direct patient care, were timed including start and end time and recorded using an adapted version of the semi‐structured audit tool created by Blay et al. (2017). To aid data collection, activities in the tool were grouped into one of the six standard categories used in workload studies (Administration, Communication, Direct care, Documentation, Indirect care, Other). Space was included in the data collection tool for activities not previously identified.

Patient data were collected from webPAS for all patients admitted on the COVID‐19 wards during the period of March–August 2021, which was compared to patients admitted during the period of March–August 2022. This comparison allowed for the exploration of changes to patient demographics and acuity levels caused by the virus.

In addition, a survey with five demographic questions and four open‐ended questions was distributed to nurses and junior doctors working during the pandemic in the COVID‐19 wards. The survey link was emailed to 198 nurses and 26 junior doctors that had worked on a COVID‐19 ward during the pandemic. The survey questions included changes to workloads, roles and responsibilities and the impact they felt this had on patient care during the COVID‐19 pandemic.

### 2.3 Data Analysis

Data analysis for the observation data, patient data and demographic survey questions was analysed using SPSS version 29. Observation data were analysed using descriptive statistics and presented in mean and standard deviation in time (presented as hours, minutes and seconds), and minimum and maximum time spent per activity.

Patient data were presented as means and standard deviations, with independent sample *t*‐tests being used to analyse the difference in patient age and length of stay between 2021 and 2022. The Fisher's exact test was used to test if there is a significant association between gender and year of admission.

The survey responses were analysed using descriptive statistics for the demographic questions and presented as mean and standard deviation. The open‐ended survey questions were analysed using content analysis.

### 2.4 Ethical Considerations

Permission to undertake this study Ethics approval was sought from the Human Research Ethics Committee (2022‐03334‐DOLEMAN) on the 19 April 2022. The opt‐out process of consent was applied to the observation component of the study due to the risk and difficulties of working on a COVID‐19‐positive ward. The survey was emailed online with a consent button to continue with the survey. Confidentiality and anonymity were maintained throughout. Data were stored in compliance with the NHMRC guidelines.

## 3 Results

### 3.1 Observational Results

In total, 12 junior doctor observations and 32 nurse observations were undertaken on the COVID‐19 only wards for a total of 87.16 h. On the non‐COVID‐19 ward, 21 junior doctor observations and 34 nurse observations were undertaken for a total of 131.5 h. See Table 1 for the mean time spent on each care activity between the COVID‐19 and non‐COVID‐19 wards for nurses and junior doctors. When comparing the mean activity time for nurses on the COVID‐19 ward spent less time on administration, less time on communication and documentation, and more time on direct care activities, hand hygiene and other care activities than nurses working on the non‐COVID‐19 ward. Junior doctors on the COVID‐19 ward spent less time on administration work and direct care activities, spent more time communicating, completing documentation, undertaking hand hygiene and other tasks compared to junior doctors working on the non‐COVID wards.

**TABLE 1:** Observational activity times for the COVID and non‐COVID wards.

Activity	COVID‐19 wards (h:mm:ss) Mean (±SD) time Min–max		Non‐COVID‐19 ward (h:mm:ss) Mean (±SD) Min–max	
	Nurse	Junior doctor	Nurse	Junior doctor
Administration (request order or service/filing/photocopying)	0:04:05 (±0:04:09) 0:00:00–0:14:20	0:04:34 (±0:04:01) 0:00:00–0:11:10	0:05:37 (±0:07:08) 0:00:00–0:32:21	0:08:03 (±0:06:27) 0:01:04–0:27:13
Communication (patients/family/nurses/medical practitioners/other health workers/other ward and students)	0:23:03 (±0:16:00) 0:00:00–0:52:51	1:09:19 (±0:34:35) 0:11:00–2:01:56	0:51:41 (±0:30:11) 0:09:20–2:51:46	0:48:52 (±0:13:56) 0:17:15–1:18:03
Direct care (patient assessment/chronic disease management/patient education, vital signs/nursing care/medication/IDC management/wound management/resuscitation/specimen collection)	0:38:02 (±0:18:50) 0:07:21–01:31:27	0:05:25 (±0:12:15) 0:00:00–0:43:04	0:37:49 (±0:21:23) 0:07:34–1:36:36	0:31:33 (±0:21:26) 0:03:55–1:22:43
Documentation (hard copy/electronic/discharge)	0:18:22 (±0:10:11) 0:00:00–0:36:27	0:48:02 (±0:14:24) 0:00:00–0:43:04	0:24:36 (±0:15:06) 0:00:00–1:07:33	0:34:28 (±0:10:52) 0:15:52–0:56:24
Hand hygiene/PPE	0:07:07 (±0:04:03) 0:00:00–0:16:10	0:04:07 (±0:03:14) 0:00:30–0:12:25	0:06:45(±0:08:30) 0:01:04–0:49:12	0:01:56 (±0:00:54) 0:00:39–0:04:33
Other (site preparation/searching for equipment/external transit/internal transit/cleaning)	0:07:36 (±0:08:17) 0:00:00–0:33:01	0:03:04 (±0:03:28) 0:00:00–0:08:30	0:07:42 (±0:11:40) 0:00:00–1:04:35	0:01:20 (±0:02:08) 0:00:00–0:09:07

### 3.2 Patient Characteristics

In total, 2056 patient records were collected from webPAS across 2021 and 2022 (*n* = 1227 for 2021 and *n* = 829 in 2022) for the COVID‐19 wards. Results indicated that patients admitted in 2022 were statistically significantly older (65.2 ± 18.59) than those admitted in 2021 (62.9 ± 18.56), t (2,024) = −2.736, *p* = 0.006. Patients admitted in 2022 had statistically significantly shorter length of stay (6.86 ± 9.94) compared to those admitted in 2021 (7.96 ± 8.74), t (2,054) =2.635, *p* = 0.008. However, most admissions were emergency during 2022 (81.5%) compared to 67.8% during 2021. The Fisher's exact test did not indicate any association between the gender of patients and the year of admission (two‐tailed *p* = 0.094). However, admission types were significantly different between 2021 and 2022. The 2022 group had more emergency admission patients (two‐tailed *p* < 0.001). See Table 2 for demographic and clinical characteristics during 2021 and 2022.

**TABLE 2:** Demographical and clinical characteristics of the patients (*N* = 2056).

Variable	Total (*N* = 2056)	2021 (*n* = 1227)	2022 (*n* = 829)	*p*
Age (M ± SD)	63.9 ± 18.60 Min = 16, Max = 107	62.9 ± 18.56 Min = 16, Max = 101	65.2 ± 18.59 Min = 16, Max = 107	0.006^a^
Gender				
Female	977 (47.5%)	599 (48.8%)	378 (45.6%)	0.094
Male	1049 (51.0%)	612 (49.9%)	437 (52.7%)	
Missing	30 (1.5%)	16 (1.3%)	14 (1.7%)	
Admission				
Elective/direct admission	543 (26.4%)	393 (32.0%)	150 (18.1%)	< 0.001^a^
Emergency admission	1513 (73.6%)	834 (68.0%)	679 (81.9%)	
LOS (M ± SD)	7.51 ± 9.25 Min = 1, Max = 176	7.96 ± 8.74 Min = 1, Max = 78	6.86 ± 9.94 Min = 1, Max = 176	0.008^a^

*Note:* % is the percentage within each group.

^a^ *p* < 0.01.

### 3.3 Survey Responses

In total, 19 surveys were returned from a total of 224 distributed (response rated 8.4%). Despite the low response rate, data saturation was reached. Of these, 17 were females, and two were male. The average age was 40 years (± 11.45). Ten (52.6%) participants worked full time, and nine (47.4%) worked part‐time. The average years in the current role was 9.26 years (min 1 year—max 43 years). The majority of responses were from registered nurses (68.4%). See Table 3 for participant demographics.

**TABLE 3:** Participant demographics.

Demographics	Mean (SD)
Age	40 (+ 11.45)
Years in role	9.26 years (min 1 year–max 43 years)
*N* (%)	
Gender	
Female	17 (89.5%)
Male	2 (10.5%)
Role	
Registered nurse	13 (68.4%)
Clinical nurse	4 (21.1%)
Medical registrar	2 (10.5%)
Work type	
Part‐time	10 (52.6%)
Full time	9 (47.4%)

### 3.4 Open‐Ended Survey Responses

The five open‐ended questions from the survey were analysed using, and five themes were identified using thematic analysis. These themes were grouped under the category of staffing shortages resulting in higher workloads, requirements to work overtime and work outside of competency, unforeseen time to undertake tasks, lack of support, mental and physical health and patient care compromised.

### 3.5 Staffing Shortages Resulting in Higher Workloads, Requirements to Work Overtime and Work Outside of Competency

Working on an understaffed shift resulted in an increase in workloads for both nurses and junior doctors. One participant explained:

Covering staff shortages, often having to take on extra work as other people were sick. Doing more on call D2

Some of the participants went on to explain that due to the staffing shortages they felt a moral obligation to work overtime:

Felt obliged (morally, not forced) to work extra shifts, including doubles as we had very few shifts where we were fully staffed N13.

In addition, the staffing shortages resulted in both nurses and junior doctors taking on roles and responsibilities that they were not competent or trained to undertake. One doctor described this in the following way:

Taking on advanced trainee consults D2.

Nurses with limited experience were also taking on roles that they did not have adequate experience to undertake. For example, a nurse went on to describe that with 2 years of experience she was the most senior nurse on the ward.

I was asked as a level 1.2 to shift co‐ordinate… given I was the most "senior" during an afternoon shift. N4

Nurses also discussed an increase in workloads caused by staffing shortage. This was discussed in relation to an increase in nurse‐to‐patient ratios. For example

Nurse patient ratio was 1.6 or 8 at times. The workload was very heavy considering most of the patients were full nursing care. N17

In addition to staffing shortages, workloads were also discussed in relation to patient demographics indicating that patients were older and required more care associated with activities of daily living. For example, one nurses described this is in the following way:

Workload was high as had influx of outbreaks from nursing home facility's…which led to high acuity of patients some shifts had numerous hoists, low staffing to support ADLs N7

And another nurse described their experience of workloads, patient‐to‐nurses ratios and acuity in the following way:

It was hard work. Very acute sick, unwell patients that need full nursing care due to illness (Covid + other co‐morbidities) …. Workload of 4 to 6 patients per shift (due to shortage of nursing staff) each day with such intensity—is very challenging N14.

### 3.6 Unforeseen Time to Undertake Tasks

Participants felt that caring for patients during the pandemic resulted in unforeseen time to undertake tasks associated with donning and doffing. One participant stated:

Very time consuming constantly donning and doffing N1.

which reduced the efficiency of care;

The addition of donning and doffing added unforeseen time into everyday tasks of nursing so it felt as though you couldn't be as efficient or responsive as normal. N11

In addition, participants felt they had limited time to complete tasks safely, focus on patient care or spend adequate time with patients, which they felt impacted on the care that was provided. One junior doctor described their experience in the following way:

We had less time to see each patient, or often saw them quite late, which may have impacted on patient care. D1

### 3.7 Lack of Support

The majority of participants felt that staff working on the COVID‐19 ward had limited work experience and a lack of mentoring. They also discussed the increase in relief nurse use in order to fill nursing workforce shortages. One participant explained this in the following way:

Too many junior and relief nurses, insufficient mentoring and staff development. N1

Participants also felt they had no education or staff development opportunities available as they took on new responsibilities and roles, which many were too junior to have developed during their careers. However, collegial relationships were identified as being important for the provision of support and guidance to junior staff during the pandemic. One nurse discussed:

I also felt that graduate nurses specifically were being given roles and responsibilities without the support of staff development nurses and the proper guidance and education that I know lead to things like medication errors and poor time management.

Although many were grateful for collegial relationships and teamwork. N4

There was limited support from other doctors and allied health staff as they were not willing or available to enter the COVID‐19 ward to look after COVID‐19 patients. Participants felt that this compromised patient care and resulted in sub‐standard care.

A number of clinical teams were reluctant to visit the COVID ward to consult on patients, compromising their care. Also provision certain services (i.e. imaging/allied health) was also sub‐standard on COVID ward patients. N10

Also,

Doctors and teams were not available as they are on regular wards, staff unfamiliar with specialities, patient been treated for covid and not for their co morbidities such as renal patients. N3

### 3.8 Mental and Physical Health

Participants felt that working during the pandemic resulted in an increase in burnout caused by staffing shortages, and the pressures of the working environment. These changes to the work environment were felt to impact the provision of patient care but also on their professional satisfaction of nurses and junior doctors. One nurse described this in the following way:

It led to burnout for myself and fellow colleagues. We also felt very frustrated with staffing and were constantly put under so much pressure and stress that did not benefit the working environment. It made it quite a hostile environment for many people. I believe that impacts nursing care and patient care delivery as our joy and desire to be great nurses was being affected. N4

And another discussed how the pressures of the environment resulted in them wanting to leave the profession altogether.

It was the most challenging experience I was faced with during my whole nursing career. Me and my colleagues were not supported in a way we should have been as we were working in an unsafe environment. There were many shifts where we did not get our breaks and stayed back longer than we had to. It was not staffed well as the majority of staff working there were junior or relieving from other areas who had no idea what they were doing. It made me want to quit nursing altogether and I'd never do it again. N5

The lack of staffing and the demands of donning and doffing also resulted in staff working without meal breaks or being able to hydrate throughout their shifts.

90% of my shifts I did not have a meal break! Not just me, all of us, and we never got paid for working non‐stop. N14

Participants also felt they were not recognised or appreciated for their roles during the pandemic and discussed the physical strain of having to wear PPE all of the time;

Lack of recognition from executive about the strain wearing full PPE had on staff. N1

The frustrations of working on the COVID‐19 wards were also discussed in terms of watching their colleague's fatigue from a lack of staffing and having to undertake different roles and responsibilities. One nurse described their experience in the following way;

Upsetting to see my colleague's fatigue, my CNS not being supported—often acting as CNS, SDN, Shift coordinator and being hands‐on with patients. Frustrated at some staff being allocated to relieve for a shift on the ward and refuse or just not turn up. N13

Respondents also discussed receiving abuse from patients and family during the pandemic which impacted negatively on mental health. For example:

Huge responsibility for staff safety, for patient safety, often being abused by patients or families over the phone, often being in Code Black due to delirium (secondary to Covid). N14.

However, participants were grateful for strong collegial relationships and teamwork during the pandemic with many feeling that they had learnt a lot. For example:

Positives: I worked with some really fantastic people who brought their great skills, from many specialities to the ward. They were invaluable because we had such a mix of patients, I learned a lot. N18

### 3.9 Patient Care Compromised

Most participants felt that patient care was compromised as they did not have time to undertake basic care needs such as showering patients or providing basic hygiene needs. This was mainly due to patient acuity on the wards and the extra support with ADLs that were required.

I believe that I, and the other staff I was working alongside, did not have the time to carry out our tasks in a safe and effective manner on some days due to time constraints and having to focus on the more "sicker" patients. N5

Another discussed the impact of high workloads on the ability of nurses to meet patient hygiene needs.

Hygiene changes weren't met very well due to high acuity of workload. N7

Patient care was further compromised by limited resources on the wards, including adequate staff and not having the skills or knowledge to look after patients who were being admitted to the wards. They also discussed the increase in, being understaffed and nurses not having the speciality knowledge to look after sick patients;

Critically understaffed ward! Critically overworked staff! Critically under‐trained staff!

Mix of clinical presentations and diagnosis that did not match staff skills/experience/knowledge… Lack of resources such as: continence pads, airway tubing and attachments, medications, consumables… N14

Participants also discussed not being able to maintain continuity of care and the provision of high‐quality patient care due to the high number of relief staff,

Due to high numbers of relieving nurses it was more difficult to ensure continuity of care… safe, knowledgeable care N1.

## 4 Discussion

This study explored the impact of the COVID‐19 public health measures on workloads, changes to direct patient care, and the impact on resource allocation during the pandemic. Data were collected through observation, WEBPAS administrative data and a survey. The COVID‐19 wards were matched to a non‐COVID ward to allow for comparison and review for any changes to workloads or patient care as a result of the COVID‐19 pandemic. The comparison of observational data showed changes to the workloads of nurses and junior doctors working on the COVID‐19 wards compared to those working on the non‐COVID‐19 ward. These findings were mirrored in the open‐ended survey responses where nurses and doctors discussed staffing shortages resulting in higher workloads and requirements to work overtime, unforeseen time to undertake tasks, lack of support, mental and physical health and patient care compromised to be present on the COVID‐19 wards. In addition, results from the review of patient data support the open‐ended responses in that patients were older. Patient characteristics also indicated that patients had shorter lengths of stay and were admitted as emergencies compared to the same period the year before. These findings are consistent with a reduction in elective admissions during COVID‐19.

One of the primary issues participants in this study described was staffing shortages, which resulted in higher‐than‐normal workloads and the need for overtime to meet patient demands. A scoping review by Doleman, De Leo, and Bloxsome (2023) and Doleman, Coventry, et al. (2023) reviewed the impact of workloads on healthcare providers during pandemics and uncovered that 54 of the 55 included studies also reported an increase in workloads. These were related to an increase in patient care needs, workload intensity, increase in overtime or hours worked per week, changes to workload responsibilities, worsened work environments and an increase in patient‐to‐nurse ratios (Doleman, De Leo, and Bloxsome 2023). When workloads are heavy, the result is an increase in negative outcomes for healthcare providers and patients including an increase in turnover, increased absenteeism, work related musculoskeletal disorders, mental health distress, increased injury and an increase in negative patient outcomes (Qureshi, Purdy, and Neumann 2018). Unfortunately, as healthcare providers leave the profession these workforce shortages persist which increases the workloads of those remaining (Lu, Zhao, and While 2019). In light of this, ‘pandemic‐adjusted staffing’ is suggested where statistical modelling and simulations are used to identify staffing numbers, working hours and epidemiological spread of disease in order to maximise staff health and operational functionality of health care facilities and system (Habib and Zinn 2020; Mascha et al. 2020). While this is useful to optimise staffing during pandemics and periods of high pressure on the healthcare sector, it is also vital that healthcare organisations asses healthcare provider workloads as we transition out of the pandemic, including having adequate breaks both on shifts and between shifts and reducing overtime where possible (Doleman, Coventry, et al. 2023). These strategies are needed to reduce the mental and physical burden of working during the pandemic which is vital for future sustainability of the workforce.

In the present study, the changes to workloads resulted in an increase in nurse and junior doctor burnout, pressure and stress, which resulted in some wanting to leave their roles. Similar results have been well documented in the literature indicating that high workloads impact mental health, causing distress in healthcare workers (Alfonsi et al. 2023; Doleman, De Leo, and Bloxsome 2023; Serrano‐Ripoll et al. 2020). In a systematic review exploring epidemic outbreaks on healthcare workers' mental health, of 117 studies reviewed it was estimated that the prevalence of acute distress disorder was highest (40%), followed by anxiety (30%), experiencing burnout (28%), depression (24%) and lastly post‐traumatic stress disorder (13%) in front line staff (2020) (Serrano‐Ripoll et al. 2020). Alfonsi et al. (2023) explored the consequences of the pandemic on nurses and physicians in Italy and reported that while both groups were negatively impacted, nurses presented a greater distress response than physicians as evident in their DASS—Depression, DASS—Anxiety and DASS—Stress scores. If staff well‐being is not supported, the mental health distress that was caused during the pandemic will continue to fuel the turnover of staff into the future. Considering the worldwide healthcare workforce shortage, healthcare organisations need to make sure staff well‐being is a top agenda item, recognising the personal impact that the pandemic has had on individual staff. Implementing system‐level support strategies, including organisational well‐being initiatives, visible leadership and co‐worker support may enhance staffs' psychological well‐being and improve job performance (Sen and Yıldırım 2023). However, we acknowledge that implementing system‐wide changes in healthcare requires addressing issues beyond the control of organisational leaders and managers. Our paper highlights the need for engagement from higher‐level stakeholders such as government agencies, trade unions and policy makers to drive effective change as the grassroots level. This approach is essential for achieving lasting improvement and cultivating a more supportive workplace environment for staff.

A concern arising from this study was the impact of the pandemic on the provision of safe patient care. This was related to limited skills of staff, staffing shortages, limited time and limited support. Our findings are consistent with other research exploring the impact of the pandemic on patient care (Bellanti et al. 2021; Bergman et al. 2021; Brophy et al. 2021; Shiao et al. 2007). In addition, some studies also indicated an unwillingness of healthcare workers to provide care to patient with COVID‐19 citing fear of passing on the virus to loved ones and those around them (Bellanti et al. 2021; Shiao et al. 2007). In addition, nurses in a study conducted by Liang, Wu, and Wu (2021) also identified that care felt depersonalised as many grouped care activities and provided care at one time instead of needing to don and doff to enter the room on multiple occasions. An earlier study by Malekpour et al. (2014) identified that nurses undertake 80% of direct patient care. As indicated by the results of our study, nurses on the COVID‐19 ward spent less time on administration and communication and more time on providing direct patient care and hand hygiene compared to the junior doctors who focused on documentation. When communication is not adequate an increase in negative patient outcomes can be identified including an increase in medication errors (Hassan 2018). In addition, the results indicated that the roles and work responsibilities of nurses and junior doctors changed during the pandemic. This was in part due to healthcare workers, that is, allied health not wanting to attend the COVID‐19 wards. Therefore, supporting staff including having adequate resources and staffing numbers needs to be reviewed during periods of high workloads, including during pandemics and seasonal flu influences. During the pandemic, the government in the United Kingdom (UK) graduated medical students early so they could commence work as a junior doctors and physicians retired within the last 3 years were asked to return to clinical practice. Staff were also moved from education and research to support the workforce in the clinical setting (Willan et al. 2020). However, there is limited research on the effectiveness of this approach on provision of safe patient care and limited information for nursing. Therefore, utilising students, and supportive care workers to provide basic patient care, needs to be explored as a way reduce the burden on nurses and junior doctors and promote quality patient care during periods of high workloads.

This study is the first of its kind to compare time spent by nurses and junior doctors on tasks undertaken during the COVID pandemic in the acute care sector. Based on the results of our study, the observed differences in the time allocation of nurses and junior doctors between COVID‐19 and non‐COVID‐19 wards offer valuable insights into the distinctive demands imposed by the pandemic. The pandemic undoubtedly placed healthcare professionals in uncharted territory, demanding resilience, adaptability and dedication. The findings in this study have delved into the myriad of challenges faced by nurses' and junior doctors in one hospital in WA, shedding light on critical issues that have far‐reaching implications for both healthcare professionals and patient care into the future.

### 4.1 Limitations

This study was conducted in one public hospital in WA and may not be generalisable to other settings. We have provided thorough description to allow for the results to be inferred to other settings. We maintained a presence in the COVID and non‐COVID wards over a period of time when permitted to increase the participation rate; however, the authors acknowledge that not all activities were observed/timed.

## 5 Conclusion

This study comprehensively examined nurses' and junior doctors' challenges during the COVID‐19 pandemic and compared the time nurses' and junior doctors spent on COVID and non‐COVID wards completing tasks such as administration, communication, direct care, documentation and indirect care. The study highlights the urgent need for system‐wide changes to the healthcare workforce, from staffing shortages and elevated workloads to compromised mental and physical health. As we collectively reflect on the lessons learned from this unprecedented period, it is imperative to address these challenges proactively, fostering a healthcare environment that prioritises the well‐being of its front line nurses and junior doctors and, by extension, the quality of patient care.

## Author Contributions

All authors contributed equally to this work.

## Ethics Statement

Approval to undertake the study was granted from the University HREC 222‐03334‐DOLEMAN.

## Conflicts of Interest

The authors declare no conflicts of interest.

## Peer Review

The peer review history for this article is available at https://www.webofscience.com/api/gateway/wos/peer‐review/10.1111/jan.16487.

## Supporting information


Data S1.

## Data Availability

Data is not available due to ethical restrictions.
